# Unraveling the Thread of Aphasia Rehabilitation: A Translational Cognitive Perspective

**DOI:** 10.3390/biomedicines11102856

**Published:** 2023-10-21

**Authors:** Georgios Papageorgiou, Dimitrios Kasselimis, Nikolaos Laskaris, Constantin Potagas

**Affiliations:** 1 Neuropsychology and Language Disorders Unit, 1st Department of Neurology, Eginition Hospital, National and Kapodistrian University of Athens, 11528 Athens, Greece; 2 Department of Psychology, Panteion University of Social and Political Sciences, 17671 Athens, Greece; 3 Department of Industrial Design and Production Engineering, School of Engineering, University of West Attica, 12241 Athens, Greece

**Keywords:** translational neuroscience, neuroplasticity, aphasia, rehabilitation, stroke, language impairment, cognitive recovery, brain, animals, language

## Abstract

Translational neuroscience is a multidisciplinary field that aims to bridge the gap between basic science and clinical practice. Regarding aphasia rehabilitation, there are still several unresolved issues related to the neural mechanisms that optimize language treatment. Although there are studies providing indications toward a translational approach to the remediation of acquired language disorders, the incorporation of fundamental neuroplasticity principles into this field is still in progress. From that aspect, in this narrative review, we discuss some key neuroplasticity principles, which have been elucidated through animal studies and which could eventually be applied in the context of aphasia treatment. This translational approach could be further strengthened by the implementation of intervention strategies that incorporate the idea that language is supported by domain-general mechanisms, which highlights the impact of non-linguistic factors in post-stroke language recovery. Here, we highlight that translational research in aphasia has the potential to advance our knowledge of brain–language relationships. We further argue that advances in this field could lead to improvement in the remediation of acquired language disturbances by remodeling the rationale of aphasia–therapy approaches. Arguably, the complex anatomy and phenomenology of aphasia dictate the need for a multidisciplinary approach with one of its main pillars being translational research.

## 1. Introduction

The principles that govern language rehabilitation remain a perpetual topic of interest in the field of aphasia [1]. In the short history of language treatment, there have been several approaches to the study of aphasia rehabilitation. Most of them usually focus on language per se, whether it is the exact aphasic profile, the type and/or severity of observed language disturbances, the underlying—and supposedly impaired—language mechanisms, or techniques to enhance verbal behavior and overall communication ability. (For a review of these approaches, see [2]). This is probably derived from the fact that for more than a century, the Wernicke–Lichtheim model defined not only the neural and functional substrate of language [3] but also the ideas and strategies concerning aphasia rehabilitation [1].

In recent years, an emerging alternative perspective, based on comparative anatomy, neuroimaging, and lesion studies, has contradicted this functional organization dogma. This theoretical perspective states that the so-called “language network” may have evolved before the emergence of language as a neural substrate of a domain-general processing mechanism [3]. Thus, language faculty could be viewed as the product of natural selection based on physiological and cognitive pre-adaptations, such as perisylvian networks or white-matter pathways that are also present in animals [4], which may appear, prima facie, to be specialized for discrete functions such as syntax but actually support other, more fundamental cognitive domains, such as working memory (for a relevant short discussion, see [5]).

For example, it has been argued that Broca’s area, a traditionally labeled “language” region that has primarily been associated with speech production, is a “supra-modal hierarchical processor”, even in non-verbal tasks (see [6]). Ιt has been demonstrated that Broca’s area is engaged in abstract sequencing [7] as well as in the processing of other types of information related to complex motor sequences, music, or mathematics [8] (for a review of the involvement of Broca’s area in several non-language processes, see [9]). There is also evidence, derived from healthy brain functioning, of the involvement of perisylvian “language” regions in a broad spectrum of executive functions. Brodmann area 45 has been shown to be involved in selective retrieval [10], while Brodmann area 46 and inferior parietal cortices have been associated with monitoring within working memory and manipulation, respectively [11,12]. 

In a similar context, there have been studies showing that the dorsal or ventral streams that are associated with language are critical in other, more “basic” cognitive functions. For example, the third branch of the superior longitudinal fasciculus which connects prefrontal, premotor and parietal areas (SLF III) is involved in phonological processing, but it is also assumed to control orofacial action, even in non-verbal tasks [13]. On the other hand, the extreme capsule fasciculus, which is also present in the macaque monkey [14], has been associated with semantic language processing, while there are studies that support its role in the actively controlled retrieval of information [15].

Lesion studies on aphasia have also shown that patients with acquired language disturbances commonly face difficulties in other cognitive domains, such as short-term memory, working memory [16,17], or other executive functions (for a review, see [18]). Overall, an aphasia-producing lesion will inevitably result in deficits in cognitive domains other than language, and these deficits have been shown to be related to the severity of language. This notion is further supported by lesion studies that do not focus on aphasia per se but investigate lesion loci that affect language-related areas. It should be noted that the latter term is not used a strict sense here, and thus it is not limited to the traditional regions identified as “Broca’s” and “Wernicke’s” areas but rather extends to a quite broad perisylvian region that includes cortices or even white matter pathways, which have been associated with various aspects of language processing, as indicated by brain-imaging studies (for a review, see [19]). In this context, there have been studies showing that such perisylvian lesion sites may affect several cognitive skills. For example, Baldo and Dronkers [20] showed that damage to the inferior parietal cortex and the inferior frontal cortex may differentially affect different components of working memory tasks. Leff et al. [21] argued that the superior temporal gyrus in the left hemisphere is a shared neural substrate for both auditory comprehension and short-term memory. Furthermore, Chapados and Petrides [22] highlighted the importance of the ventrolateral prefrontal cortex for selective retrieval. This notion was further supported by a recent study which showed that a lesion specifically affecting fundamental components of the ventral “language” stream, including pars triangularis and the temporo-frontal extreme capsule fasciculus, has detrimental effects on lexico-semantic processing and active selective controlled retrieval [23].

Regarding these advances that delve even more deeply into the neurobiology of language and, ultimately, raise doubt about the traditional dogma of the neural organization of language, there has been, in recent years, an ongoing debate regarding how (or even if) findings from basic neuroscience studies can be exploited in order to optimize language treatment [24]. In this vein, neuroscience research has revealed a universal characteristic of human and animal brain—neuroplasticity—which potentially serves as a bridge between basic research and clinical practice [25,26]. This emerging field, i.e., cognitive neurorehabilitation, is founded on a set of specific neural principles that could probably be translated and applied to human recovery from language and cognitive deficits [27].

**Figure 1:**
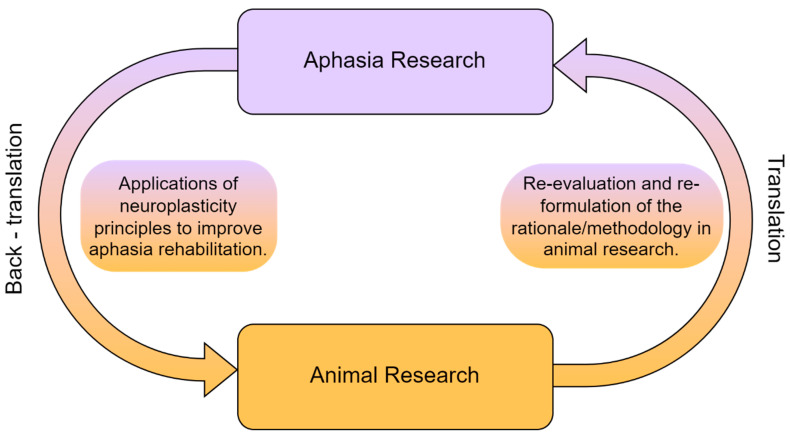
The reciprocal relationship between animal and aphasia research.

This translational approach in rehabilitation inevitably leads to two major questions. The first question is whether clinicians specialized in the rehabilitation of cognitive disorders, and particularly aphasia, can manipulate the principles of neuroplasticity in order to maximize language treatment, based on findings from animal research. The second question is broadly related to the possible links between language and other cognitive domains. Animal studies usually examine sensory and motor functions, but there are also sparse data on cognitive functions such as object recognition or spatial memory [28]. From that perspective, it is essential to take into account the idea that the grounding evolutionary foundations for language to root were probably other domain-general cognitive mechanisms [3]. Consequently, the second question is formulated as such: are there studies with stroke-induced aphasia patients which designate the significance of non-linguistic functions in language rehabilitation? In the following sections of this paper, we will attempt to describe a potential translational framework in aphasia rehabilitation (see Figure 1).

## 2. Neuroplasticity in Animals and Aphasia Research

Several animal studies in the broader field of evolutionary biology confirm that mammalian species demonstrate differences but also substantial similarities in cerebral organization and function [29]. Based on this line of research, a fundamental attribute of the brain has emerged, i.e., neuroplasticity. This term refers to the neurons’ intrinsic capacity to reorganize their structure and function in response to environmental stimuli and injuries [30]. It is well documented that humans have a larger cortical surface area compared to other animals; however, this is not the primary impetus of brain plasticity [29]. In their seminal paper, Rockel et al. [31] compared specific properties of cortical neurons such as number and density, in cat, macaque, rat and human. They concluded that the core difference across the aforementioned species was not the distribution of neurons in each section but instead the pattern of synaptic connections among brain areas. Based on that notion, it has been theorized that the ability to ‘sculpt’ these connections is the cornerstone of neuroplasticity and, more interestingly, the underlying mechanisms of this neural modification are parallel between humans and animals [32]. This hypothesis has formulated the basis of translating results from animal research to humans [25]. In general, neuroplasticity is a dynamic process underlying normal development or learning, and it includes various atrophic and trophic processes, such as neurogenesis, synaptogenesis, and the removal of unused synapses [33]. In this context, neuroscientific research has suggested that the refinement and alteration of behavior via neuroplasticity is primarily influenced by a wide variety of stimuli and experience [34]. Similar studies have indicated structural alterations in brain areas following cognitive training in animals and humans [35,36]. As Turkstra and colleagues [26] have highlighted, ‘there is an ongoing process of modification in both directions: experience to brain and brain to experience’ (p. 604). On the grounds of this interaction, it has been argued that structural mechanisms underlying experience-dependent plasticity in the cortex, such as axonal sprouting or the growth of new dendritic spines, could be manipulated toward the reorganization of cognitive functions and language following stroke [36]. Thus, the study of the principal rules governing neuroplasticity in the intact or the injured brain of both animals and humans could provide valuable guidelines for understanding how the neural circuits are remodeled following stroke either during the course of recovery or in the context of rehabilitation.

In the case of aphasia, there is accumulating evidence suggesting that spontaneous neuroplastic brain changes following stroke could result in language reorganization [36]. In general, neuroimaging studies indicate that the compensation for impaired language functions relies on the increased activation of residual undamaged left hemispheric areas or the recruitment of homologous right hemispheric areas [37]. For instance, Fridriksson [38] showed a correlation between improved naming performance and increased cortical activation in left undamaged areas in untreated post-stroke aphasia. On the other hand, patients with aphasia (PWAs) have been shown to exhibit a right-lateralized activation pattern during a silent word-generation task, which is a pattern similar to that of left hemispheric regions of healthy right-handed individuals [39]. It should be however noted that right hemisphere changes have also been reported to be maladaptive, and increased activation in those areas could be associated with impaired performance [40].

The involvement of neuroplasticity in language reorganization has been addressed not only as an important aspect of spontaneous recovery but also in the context of rehabilitation research. Although sparse, there are functional imaging studies which have demonstrated brain changes as a result of treatment programs. Thompson et al. [41] have shown that training in producing specific sentence structures may result in increased right-hemisphere activity during verb production in PWAs; the sites of such increased activation were different from those usually identified in neurologically intact individuals. Therefore, these results provided indications of remapping language functions to previously uninvolved brain regions, such as the superior parietal cortex. Furthermore, in a study by Fridriksson [42], twenty-six left stroke survivors received an intense aphasia treatment focusing on object naming. The results showed that even though damage to the left middle temporal lobe and the temporal–occipital junction had a negative effect on performance, increased brain activation in the anterior and posterior regions of the left hemisphere was correlated with improved outcomes. There are also findings highlighting treatment-induced activity changes in brain connectivity patterns involving language-related tracts, such as the arcuate fasciculus [43]; however, this line of evidence is still inconclusive [44].

Apart from functional changes, there have also been sparse reports of structural brain alterations following language rehabilitation. One study found an increase in the number of fibers and volume of the right arcuate fasciculus after melodic intonation therapy in PWAs [45]. It has also been shown that an improvement of word retrieval may be associated with increased structural integrity of the left arcuate fasciculus [24]. Furthermore, improved naming performance has been associated with different patterns of gray matter density in specific right hemisphere areas, such as the precentral gyrus or the temporal lobe [44]. A study by Allendorfer et al. [46] reported increased axonal density in left frontal areas following transcranial magnetic stimulation over the left hemisphere; nevertheless, more research is required to clarify the effect of gray and white matter changes on specific language domains. 

**Figure 2:**
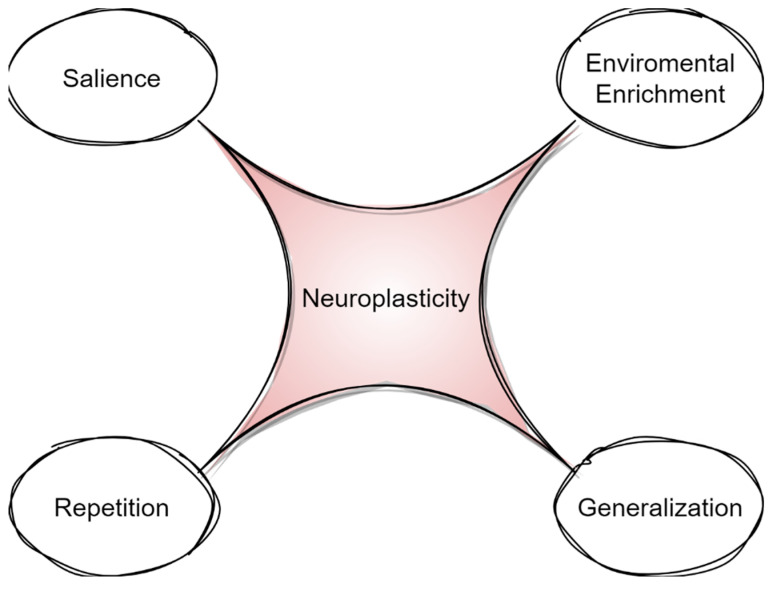
Principles of neuroplasticity.

In summary, a surge of basic and neuroimaging research indicates that neuroplasticity is the cornerstone of cognition and language recovery after brain damage. However, only recent studies have focused on specific principles of neuroplasticity that could be manipulated in order to maximize language treatment (Figure 2) [30].

## 3. Generalization, Environmental Enrichment, and Salience in Rehabilitation

In the last few decades, animal research has suggested that specific rehabilitation principles promote neuroplasticity and functional recovery [30,47]. Sparse experiments have demonstrated that treatment focused on one particular function can generalize to the improvement of untrained behavior in animals [48]. For example, Liu et al. [49] have shown that cognitive training in rats via a T-shaped maze may improve memory after a 4-week program; that improvement was accompanied by enhanced functional activity of the hippocampus and the medial–prefrontal cortex. In a similar context, there has been evidence of increased dendritic patterns in both hemispheres of rats following sensory-motor intervention during a skilled one-paw reaching task, which was also ‘transferred’ to reaching with two paws [50]. Other researchers have proposed that such generalization could be influenced by the complexity and richness of training surroundings [51]. In animal research, environmental enrichment generally refers to a more challenging environment (e.g., group housing, toys, diverse food), and it facilitates neurogenesis and synaptic plasticity [52]. It has been also argued that a more complex intervention environment may affect memory and learning. Hamm et al. [53] have shown that the training of rats in an enriched environment may result in better performance regarding spatial memory, while other studies have highlighted the recovery of motor coordination [54]. Moreover, enriched environments are considered to promote salience, which is an important factor of neuroplasticity [30]. Salience is the perceived value or relevance of the experience to the individual [27] and has been associated with motivation and attention in animals [55]. Animal research using auditory tunes has demonstrated that there could be an alteration and reorganization of auditory maps in rats when training is salience based [56].

Based on the aphasia literature, the generalization of language treatment has been a perennial issue for clinicians [57]. The implications for language reorganization is that training a specific language modality could influence the neural capacity to improve in other untrained language behaviors [30]. Several studies have examined generalization effects in other language functions when rehabilitating confrontation or picture naming (for a review, see [58]). Hillis and colleagues [59] have reported significantly better semantic and comprehension performance following naming rehabilitation, although there are approaches which doubt the methodological processes that lead to generalization gains [60]. In the domain of syntax and speech production, the training of sentences could result in generalization gains of untrained sentences when they exhibit similar grammatical and semantic properties [61]. On the other hand, the importance of salience has not been systematically studied in the field of aphasia rehabilitation. However, it is well known that PWA may demonstrate a lack of motivation in daily activities and even depression, especially when language disturbances are severe [1]. A recent study that could shed light on this subject is that of Janssen et al. [62]. The authors designed an enriched environment in a rehabilitation setting with stroke patients. The primary outcome was that patients in the enriched environment had higher engagement compared to the control group (rehabilitated in a non-enriched environment), and they also demonstrated improvement in cognitive functions. The principle of salience in aphasia should be further investigated with intervention protocols that promote motivation and are meaningful for the participant [24]. 

## 4. *Repetitio Est Mater Studiorum* or “Repetition Influences Recovery”

There are animal studies which support the idea that the training and acquisition of a learned behavior after brain injury is not sufficient for the reorganization of function [63]. Research on the principles that facilitate neuroplasticity highlights repetition and intensity as key elements for the maintenance of neural changes in the brain [64]. For instance, Monfils and Teskey [65] have reported that an increase in synaptic strength and number can be observed in rats only after several days of training. In addition, a motor map reorganization can be achieved in rats after an intense and repetitive training program [63]. However, there is still no gold standard concerning the number or the duration of trials that animals should undertake in order to achieve improved functional outcomes [24,25]. Microstimulation and functional mapping studies have also shown that repetitive exercise can influence the activity of neural circuits (for a review, see [66]). Repetitive motor training combined with brain stimulation could lead to functional improvements by reducing activity in specific brain areas [67]. It is noteworthy that repetition and intensity, although theoretically distinct principles of neuroplasticity, are often not separated in animal studies [24,68]. However, some studies have proposed that exaggerated intensity and repetition of training in rehabilitation could lead to tissue loss and reduced functional gains [69]. 

Based on these animal studies, aphasiologists have examined the issue of intensity in language treatment [70]. Greater intensity of rehabilitation, when reported, is shown to have positive functional outcomes for PWA in naming [71] or spoken language [72]. In a similar vein, there are studies which have reported an improvement of language following treatment of 8.8 h per week for 11.2 weeks [73], while others do not confirm such a positive effect [70]. It has also been noted that intensity may have positive effects on language-related functional and structural reorganization: Meinzer et al. [74] have shown increased activation in perilesional areas in PWA after an intensive 2-week training program, while Schlaug, Marchina, and Norton [45] have reported increased volume of the arcuate fasciculus after a longer intensive rehabilitation program.

In summary, the existing studies on humans, although scarce, have provided indications about the benefits of intensity; however, similarly to animal research, the specifics of such programs are yet to be fully understood [70]. Future studies should provide guidelines for the optimized duration of intervention protocols, focusing on specific language domains of PWA. 

## 5. Rehabilitation of Cognitive Functions and Its Reflection to Language

It has already been established that sensory-motor and memory functions in animals can be improved following neurorehabilitation protocols [34]. Until the field of translational research expands further, researchers can only formulate theories about possible parallels between humans and other animals concerning the structural and functional mechanisms involved in rehabilitation [25,27]. Within this context, the notion that language is supported by ‘basic’ cognitive domains (e.g., action, memory, etc.) has led scholars to investigate if the rehabilitation of non-linguistic functions also present in animals can optimize language treatment. This idea is supported by researchers who explore the critical aspect of cognitive mechanisms in the rehabilitation of language in humans [75]. 

Over the years, the elucidation of the brain–language relationship has proven to be a Sisyphean task, which is mainly due to the lack of a robust consensus for creating an accurate and comprehensive functional neuroanatomy model [76]. This nebulous picture has also affected recovery studies which primarily focus on impaired language modalities and their neural substrates and eventually ignore or underestimate the impact of non-linguistic factors on the behavioral manifestation of aphasia [77].

The idea that other cognitive mechanisms, which are obviously present in animals, can contribute to the structural and functional reshaping of neural networks supporting language is not new [78]. In recent years, there has been growing support of the notion that PWA exploit various cognitive functions for language processes, including—but not limited to—short-term or working memory [79,80], attention [81,82] οr other executive functions [83], and praxis [84]. 

This rationale has paved the way for the investigation of the presumable interrelation between attention and language recovery in PWA. Perhaps the most intriguing observation supporting this relationship is that the majority of these training studies have shown that subcomponents of attention, e.g., sustained or divided, may affect access to lexical representations [85]. Helm-Estabrooks, Connor, and Albert [86] have developed a rehabilitation program consisting of different non-verbal simple or complex attention alteration tasks. Their results have shown a significant improvement as well as generalization effects on auditory comprehension and visual analytic reasoning. There have been also findings indicating neural changes in attention pathways following language treatment [87], with increased connectivity on parietal regions of the default mode network associated with naming gains. Beyond the attention domain, early lesion studies have revealed that short-term (STM) and working memory (WM) may share common neural substrates with language [20]. This notion has been further supported by subsequent studies which have shown that it is an aphasia-producing lesion—rather than any left-lateralized lesion—that leads to STM/WM deficits [88]. In this framework, one could arguably ask whether language recovery outcomes may be affected by training verbal STM and/or WM. For instance, in their case study, Koenig-Bruhin and Studer-Eichenberger [89] reported an improvement in the delayed recall of nouns and sentences following intervention in STM and WM. It has been also suggested that reduced memory span, which is usually accessed by repetition tasks, is strongly correlated with lexical deficits and increased aphasia severity [16]. Another piece of evidence that further fortifies the argument that non-linguistic functions are of essence is that there have been studies highlighting the prognostic value of cognitive factors in language recovery [90]. For example, Gilmore, Meir, Johnson and Kiran [91] have reported that WM, inhibition and processing speed predicted language improvement in PWA, following naming and sentence comprehension rehabilitation, whereas visual STM was associated with the maintenance of naming gains after a 12-week no-treatment phase.

## 6. Discussion

As stated before, the short history of aphasia rehabilitation [1] has demonstrated that treatment strategies in general have been significantly influenced by the presumed neurobiological model for language of a particular time period, while neuroplasticity has been highlighted as an important rehabilitation factor only recently. The Wernicke–Lichtheim paradigm has been severely doubted by more recent theoretical accounts based on accumulating research evidence derived from studies involving patients with aphasia, but it has not yet been completely replaced [76] by other, more concrete, and modern language models which focus on neural language networks [92]. In this context, as has been thoroughly described in the previous section, it is undeniable that aphasiologists have only recently started to focus on the impact of fundamental cognitive functions in language therapy [78]. However, it is also undeniable that we have yet to delineate an integrated framework of aphasia rehabilitation. This could be attributed to limited research focus on the neural bases of spared, non-linguistic functions and the implementation of neuroplasticity principles (derived from animal studies) as well as their interaction with recovery variables which are essential in therapy strategies.

In general, post-stroke aphasia studies have examined the impact of clinical and demographic factors on language recovery, which are theorized to differentially affect brain plasticity [93]. In the past few years, there have been several inconsistencies concerning the influence of demographic factors such as age, sex and educational level on language spontaneous recovery or rehabilitation induced by intervention programs not only in the chronic but also in the acute or subacute phase (for a review, see [94]). It is generally accepted that younger brains have greater plasticity and ultimately a greater capacity for recovery [50]. Accordingly, it has been assumed that younger patients are more likely to recover than older patients [95]. However, more recent studies have not found a significant association between age and recovery (see for example [96]). Future research is thus required in order to thoroughly investigate and hopefully clarify the specifics of the process by which older adults with acquired aphasia demonstrate different patterns of recovery and reorganization compared to younger patients, and also how age interacts with other predictors of recovery, such as motivation or personality traits [24]. On the other hand, most researchers have confirmed an inverse relationship between recovery and lesion size, while lesion location has been shown to be rather more critical [97,98]. The degree of white-matter integrity, in both the left and right hemisphere, has also been documented to affect language rehabilitation [24]. Diffusion tensor imaging techniques have revealed that the disruption of specific white matter tracts of the left cerebral hemisphere such as the arcuate fasciculus or the superior longitudinal fasciculus may lead to speech production impairment [59]. However, there is still limited data regarding how rehabilitation methods can ‘reformulate’, structurally or functionally, specific white matter pathways. In sum, it is crucial to understand how aphasia-producing lesions may affect other cognitive domains (keeping in mind that language-related neural networks are not language specific and may be involved in other aspects of cognition), how neuroplasticity principles (repetition, environmental enrichment, generalization) may mediate observable post-stroke language recovery, and how neuroplastic mechanisms may interact with demographic, lesional, cognitive, or other variables [27].

Despite the interrelation between language and other cognitive domains, there have been sparse studies exploiting the key elements which facilitate brain plasticity in specific language modalities, such as word finding or auditory comprehension in the translational field (for a review, see [99]). In addition, the available findings regarding the impact of neuroplasticity in the enhancement of non-linguistic factors are still very limited. Thus, more data are needed in order to create efficient intervention protocols that focus on specific language domains. There have been some recent efforts, such as Semantic Feature Analysis or Phonomotor Treatment, which target the mental lexicon and phonological speech sounds, respectively; however, this line of research is still in its infancy [100,101]. Although the clinical relevance of rehabilitating specific functions is undoubted, the complexity of language material in aphasia treatment has also been shown to be beneficial in several domains such as syntax or lexical semantic impairments [61]. There have also been studies which explore the effect of non-language behaviors in aphasia recovery. For example, there have been promising results which demonstrate that rhythm and melodic intonation may lead to structural changes in the right hemisphere [45], while intention treatment has been reported to improve word retrieval following left-hand movements [102]. However, this is a field which has not been sufficiently studied. Given the potential to improve recovery outcomes with non-invasive and cognitively oriented methods, further research is required; such research attempts could focus on the neuroplasticity-induced structural and functional brain changes. 

As the field of neurorehabilitation progressively unfolds, more and more researchers are recognizing the importance of the key parameters of neuroplasticity and the critical need for the design of a neurobiological approach to aphasia therapies [27]. Animal models allow analysis of brain injuries and strokes at a molecular level and may thus provide insight to the core mechanisms of functional recovery [26]. 

In the context of this ongoing effort, researchers have developed stroke models; however, these are limited to motor recovery [103]. In this translational continuum, future animal studies should be more reflective of human cognitive deficits and recovery, while clinicians and aphasiologists could apply concepts derived from basic neuroscience more systematically [36]. In relation to the latter issue, throughout the history of post-stroke aphasia rehabilitation, important variables that facilitate neuroplasticity, such as intensity or timing of treatment [99], were often disregarded or characterized by a significant degree of variability among patients [1]. It has been recently reported that a higher intensity of treatment protocols may induce neuroplasticity, which eventually may lead to improved language outcomes [104]. Moreover, the issue of the timing of therapy deliverance has been revealed to be critical for rehabilitation protocols, since early intervention could be either beneficial or maladaptive [105]. However, more research is necessary to understand the interaction between intensity and timing of rehabilitation across different stages of recovery as well as the optimization of neural mechanisms which respond to treatment schedules.

Except for neuroimaging advances, which in the last decades can identify structural and functional changes following language treatment, the rise of neuromodulation technologies such as transcranial direct current stimulation and repetitive transcranial magnetic stimulation has allowed the immediate manipulation of training-induced neuroplasticity [44]. This effect can be achieved by facilitating activity in brain regions or by suppressing maladaptive neural processes [106] and is also combined with behavior treatment [44]. These stimulation methods have also been applied to modulate specific language domains, such as naming, even before intervention, with quite promising results [44,107]. Recent meta-analyses have suggested that the aforementioned neurostimulation techniques may also be associated with the timing of intervention, as positive treatment outcomes have been indicated in both subacute and chronic patients with aphasia [44]. However, there is still a lack of consensus with regard to the optimal choice of neuromodulation method depending on the possible implications posed by lesion size or location [108].

Even though scholars working on language rehabilitation have achieved a significant theoretical and practical development, translational aphasia research is still at its origins. Overall, the present review aimed to highlight basic principles stemming from the evidence available in the animal and human literature, in a translational framework, focused on aphasia rehabilitation. However, translational research is not a panacea and still remains rather challenging regarding not only aphasia rehabilitation but also other fields of neuroscience (for a review, see [109]). We are aware of the main impediment to this aim, i.e., the major difficulty of translating findings from animal studies to human patients with aphasia. This difficulty can be attributed to obvious reasons: brain differences between human and non-human mammals and, most importantly, the uniqueness of language in *Homo sapiens*. However, we argue that there are possible reciprocal gains from this effort: the field of aphasiology could benefit from basic neuroscience and, in turn, animal research could be inspired from the field of language treatment, thus forming a new translational direction in aphasia rehabilitation.

## 7. Conclusions

This study has highlighted findings derived from animal and aphasia research that could influence future studies in developing neurorehabilitation approaches emphasizing the improvement of cognitive factors and their reflection on language modalities based on neuroplasticity optimization. From a contemporary neuropsychological perspective, we argue that people with aphasia should not be treated as “aphasics” but as stroke patients with prominent language difficulties as well as significant deficits in other cognitive domains, which, in turn, may contribute to—or even be the root of—their language impairment. More and more researchers are recognizing the need for a holistic approach in aphasia rehabilitation; however, further progress is required in deciphering common parallels between animals and humans. This rationale, combined with treatment protocols that focus on the enhancement of neuroplasticity, via specific neural principles, and their association with language and non-language domains, could provide an innovative, neurobiological, and multi-modality foundation for aphasia rehabilitation.
